# SVMVGGNet-16: A Novel Machine and Deep Learning Based Approaches for Lung Cancer Detection using Combined SVM and VGGNet-16

**DOI:** 10.2174/0115734056348824241224100809

**Published:** 2025-01-03

**Authors:** Mohd Munazzer Ansari, Shailendra Kumar, Md Belal Bin Heyat, Hadaate Ullah, Mohd Ammar Bin Hayat, Sumbul, Saba Parveen, Ahmad Ali, Tao Zhang

**Affiliations:** 1 Department of Electronic and Communication Engineering, Integral University, Lucknow, India; 2 CenBRAIN Neurotech Center of Excellence, School of Engineering, Westlake University, Hangzhou, Zhejiang, China; 3 Department of Electrical and Electronic Engineering, University of Science and Technology Chittagong, Chittagong, Bangladesh; 4 College of Intelligent Systems Science and Engineering, Harbin Engineering University, Harbin, China; 5 Department of Ilmul Qabalat wa Amraze Niswan, University College of Unani, Tonk, Rajasthan, India; 6 College of Electronics and Information Engineering, Shenzhen University, Shenzhen, China; 7 College of Computer Science and Software Engineering, Shenzhen University, Shenzhen, China; 8 School of Life Science and Technology, University of Electronic Science and Technology of China, Chengdu, Sichuan, China

**Keywords:** AI, Medical machine learning, Deep learning, Image classification, Cancer, Healthcare, Disease, Diagnosis, Bio-imaging, Medical intelligence

## Abstract

**Background and Objective:**

Lung cancer remains a leading cause of cancer-related mortality worldwide, necessitating early and accurate detection methods. Our study aims to enhance lung cancer detection by integrating VGGNet-16 form of Convolutional Neural Networks (CNNs) and Support Vector Machines (SVM) into a hybrid model (SVMVGGNet-16), leveraging the strengths of both models for high accuracy and reliability in classifying lung cancer types in different 4 classes such as adenocarcinoma (ADC), large cell carcinoma (LCC), Normal, and squamous cell carcinoma (SCC).

**Methods:**

Using the LIDC-IDRI dataset, we pre-processed images with a median filter and histogram equalization, segmented lung tumors through thresholding and edge detection, and extracted geometric features such as area, perimeter, eccentricity, compactness, and circularity. VGGNet-16 and SVM employed for feature extraction and classification, respectively. Performance matrices were evaluated using accuracy, AUC, recall, precision, and F1-score. Both VGGNet-16 and SVM underwent comparative analysis during the training, validation, and testing phases.

**Results:**

The SVMVGGNet-16 model outperformed both, with a training accuracy (97.22%), AUC (99.42%), recall (94.22%), precision (95.28%), and F1-score (94.68%). In testing, our SVMVGGNet-16 model maintained high accuracy (96.72%), with an AUC (96.87%), recall (84.67%), precision (87.40%), and F1-score (85.73%).

**Conclusion:**

Our experimental results demonstrate the potential of SVMVGGNet-16 in improving diagnostic performance, leading to earlier detection and better treatment outcomes. Future work includes refining the model, expanding datasets, conducting clinical trials, and integrating the system into clinical practice to ensure practical usability.

## 1 INTRODUCTION

Globally, lung cancer is recognized as the deadliest and most devastating types of cancer [1-4]. Detecting the lung cancer proves to be considerable challenge, as its symptoms typically manifest only in the later and advanced stages [5]. Yet, the prospects of reducing the mortality rate associated with lung cancer hinge on early detection and appropriate treatment provision of patients [6]. While lung cancer primarily originates within the lungs, it occasionally presents early symptoms before spreading [7]. Recent years have witnessed the development of numerous techniques and on-going research endeavours aimed at effectively identifying lung cancer [8]. Among these techniques, CT scan images emerge as the foremost imaging modality for early diagnosis [9, 10]. Nevertheless, interpretation and detection of cancer from CT scan images can pose a formidable task for healthcare professionals [11, 12]. Fig. (**1**) provides the projected statistics for recently identified cancer cases and fatalities in the U.S., categorized by cancer site. These data includes both comprehensive projections and gender-specific details for different cancer types in the year 2023 [13]. These estimations extrapolated from information supplied by the North American Association of Central Cancer Registries (NAACCR) (https://www.naaccr.org/cancer-in-north-america-cina-volumes/ accessed on 04 March 2024) [14]. Furthermore, the predictions of impermanence are based on mortality data from the U.S. collected in the year 2006 and 2023, as reported by the National Centre for Health Statistics (NCHS) [13] and the Centres for Disease Control (https://www.cdc.gov/ mmwr/volumes/73/wr/mm7331a1.htm accessed on 04 March 2024) and Prevention (CDCP) [15]. As per the American Cancer Society (ACS) (https://seer.cancer.gov/statfacts/ html/common.html accessed on 04 March 2024), it observed that lung cancer exhibits a higher fatality rate compared to all other forms of cancer, resulting in an estimated global death toll of approximately 0.13 million individuals [16]. Every year, a substantial number of fresh instances of lung cancer are identified, with an anticipated 0.237 million fresh instances in 2023. The elevated fatality percentage is linked to the advanced stage at which this cancer is commonly identified, resulting in a higher proportion of new cases to fatalities compared to other forms of cancer [17].

Lung cancer manifests in various histological forms, encompassing ADC originating in the glandular cells of the lungs, LCC identified by the presence of large, abnormal-looking cells, and SCC arisen from the thin, flat squamous cells resembling fish scales [18]. Specifically, ADC, prevalent on the lung's outer surface, stands as the most frequent cell type in lung cancer cases. LCC undifferentiated distinguished by its swift growth, can occur anywhere within the lung. Conversely, SCC is closely associated with smoking and typically arises in the central lung region [19]. In the domain of detecting lung cancer, the VGGNet-16 emerged as a pivotal tool, renowned for its proficiency in image analysis and classification tasks [20]. Leveraging its deep architecture, VGGNet-16 autonomously extracts intricate features from chest radiographs, discerning subtle patterns indicative of lung malignancies [21]. With layers meticulously designed for hierarchical feature abstraction, it effectively captures morphological nuances specific to ADC, LCC, SCC, and normal lung tissues [22]. The VGGNet-16’s adaptability and robustness in processing medical images empower clinicians with accurate diagnostic insights, revolutionizing lung cancer screening and treatment strategies [23].

Within the sphere of cancer detection, treatment prognosis, and enhancing post-diagnosis patient survival, a diverse array of methods is currently under exploration [24]. Medical professionals and researchers employ various techniques to facilitate early cancer identification, evaluation, and classification [25]. In contemporary healthcare, ML models are of utmost importance in the identification of patterns, analysis, and categorization of critical medical conditions [26, 27]. Among these models, CNNs have surfaced as a particularly auspicious pathway for the timely identification, surveillance, and categorization of lung cancer *via* the scrutiny of the CT-scan images [28].

### 1.1 Challenges in the Lung Cancer Research

To effectively situate our scholarly contributions, it is imperative to elucidate the challenges identified in prior literature regarding lung cancer detection, particularly those that our research endeavors to address. Below are several significant challenges from antecedent investigations that our study is poised to overcome:

#### 1.1.1 Restricted Multi-class Classification

Earlier research primarily dealt with binary classification (such as cancerous *versus* non-cancerous), which unfortunately resulted in the neglect of the distinct lung cancer subtype differences [29]. This limitation constrains their applicability in clinical contexts where the comprehension of cancer subtypes is of paramount importance. Our research remedies this deficiency by devising a model that classifies four distinct types: ADC, LCC, normal, and SCC.

#### 1.1.2 Dependence on a Single Model Architecture

Numerous prior studies have been anchored in either CNNs or traditional ML models such as SVM, resulting in constraints regarding feature extraction or classification accuracy [30]. While CNNs are adept at feature extraction, they may exhibit overfitting without appropriate generalization; conversely, SVMs are proficient in classification but may lack the intricate feature extraction necessary for image data. Our hybrid (SVMVGGNet-16) model synthesizes the advantages of VGGNet-16 and SVM, effectively surmounting these impediments by harnessing CNNs for feature extraction and SVM for robust classification.

#### 1.1.3 Inadequate Image Pre-processing

Previous investigations often relied on simplistic pre-processing methods, which may neglect critical tumor features or inadequately standardize image quality. This can result in inaccurate segmentation and diminished model reliability [31]. In contrast, our investigation adopts sophisticated pre-processing methodologies, encompassing median filtering, histogram equalization, and edge detection, which collectively enhance image quality and precision in feature extraction, thereby improving model accuracy.

#### 1.1.4 Lack of Robust Performance Metrics

Earlier studies often relied on singular or limited performance metrics, such as accuracy alone, for model evaluation [32]. However, accuracy in isolation may not reliably reflect genuine performance, particularly in the context of imbalanced datasets. Our research employs a comprehensive array of metrics, including AUC, recall, precision, and F1-score, to furnish a holistic evaluation of model efficacy and reliability.

#### 1.1.5 Limited Clinical Applicability

Numerous antecedent models demonstrated commendable accuracy but exhibited deficiencies in generalizability within clinical settings due to inadequate testing on diverse patient populations or real-world validation. Our work not only achieves elevated performance but also delineates future trajectories, including the expansion of datasets, the execution of clinical trials, and the integration of the model into practical clinical workflows, thereby establishing a foundation for real-world applicability [33].

#### 1.1.6 Challenges in Data Generalization and Overfitting

Previous investigations frequently encountered challenges related to overfitting and struggled to generalize to novel datasets due to restricted sample sizes or limited feature diversity [34]. By incorporating rigorous training, validation, and testing phases, our research effectively addresses these concerns, presenting a model that sustains performance across a spectrum of diverse datasets [35].

These challenges elucidate the limitations inherent in the existing literature and furnish a robust framework to underscore the enhancements and contributions that our research imparts to lung cancer detection.

### 1.2 Motivations of the Study

The motivations of our study are as follows:

- The main drive behind this research addresses the urgent need for early detection of lung cancer, which greatly affects patient outcomes and survival rates, by creating a sophisticated and reliable detection system.
- Additionally, the research aims to enrich the domain of medical image analysis by integrating cutting-edge DL and ML techniques to enhance the accuracy of automated systems in diagnosing lung cancer.
- The study also seeks to evaluate the performance of the proposed SVMVGGNet-16 model against current methodologies, showcasing the progress and efficacy of the new approach in enhancing the accuracy of lung cancer detection and supporting medical practitioners in clinical decision-making.

### 1.3 Objective of the Study

The objective of our study certainly condensed into three points:

- The primary objective of the study involves establishing a reliable and accurate automated system for the early detection of lung cancer, with an emphasis on classifying lung nodules using advanced DL (deep learning) and ML (machine learning) techniques. Our examination evaluates and compares the efficacy of VGGNet-16 and SVM for initially detecting lung cancer using CT scan images across various stages of training, validation, and testing.
- The research also entails the integration of VGGNet-16 and SVM into an SVMVGGNet-16 model to leverage the advantages of both DL and ML techniques, aiming to enhance classification accuracy and robustness in the identification of various classes such as ADC, LDC, Normal, and SCC of lung cancer
- Furthermore, a comprehensive comparative evaluation of the proposed models will be carried out using the LIDC-IDRI dataset to assess their performance across various metrics such as accuracy, AUC, recall, precision, and F1-score and determine the most efficient approach for lung cancer detection.

### 1.4 Main Contributions of the Study

To elucidate the originality and contributions of our research, it is advisable to concentrate on the following dimensions:

#### 1.4.1 Hybrid Model Innovation

It is imperative to underscore that our research introduces a pioneering hybrid model that amalgamates VGGNet-16, CNN with SVM, thereby uniting the feature extraction capabilities inherent in DL with the classification precision characteristic of SVM. This dual-faceted methodology enhances the efficacy of lung cancer detection by effectively capturing intricate imaging features while concurrently mitigating classification inaccuracies [12].

#### 1.4.2 Classification of Multiple Lung Cancer Types

In contrast to numerous studies that predominantly concentrate on binary classification or a singular cancer type, our investigation engages with the differentiation of four distinct lung cancer classes—ADC, LCC, normal, and SCC [36]. The emphasis on this multi-class framework illustrates the adaptability of our model in addressing various subtypes, thereby furnishing a more holistic diagnostic instrument.

#### 1.4.3 Advanced Pre-processing and Segmentation Techniques

Clearly outlining our use of sophisticated techniques like median filtering, histogram equalization, thresholding, and edge detection is necessary for effective tumor segmentation. These methodologies enhance the robustness of our model and facilitate superior feature extraction, which is vital for achieving precise classification and early detection [2].

#### 1.4.4 Superior Performance with Rigorous Evaluation Metrics

The outcomes of our research exceed current benchmarks regarding training and testing accuracy, AUC, recall, precision, and F1-score. Highlighting this enhancement in performance emphasizes the credibility and utility of our model, distinguishing it from traditional diagnostic methodologies [37].

#### 1.4.5 Potential for Clinical Application

It is critical to note that our study effectively bridges the chasm between computational models and clinical application, thereby facilitating integration into practical medical workflows. Emphasizing prospective future endeavors, such as the expansion of datasets and the initiation of clinical trials, illustrates the potential ramifications of the study in the realm of lung cancer management [38].

These elements accentuate the contributions and innovative facets of our research, distinctly differentiating it from extant studies and underscoring its practical relevance.

### 1.5 Organization of the Study

Our paper encompasses several key sections essential for a comprehensive exploration of lung cancer detection methodologies. It commences with an introduction, setting the stage by elucidating the significance of initially detecting lung cancer and the necessity for advanced computational approaches. Section 2 deals with thorough related work follows, synthesizing existing research on the topic, elucidating various methodologies, and highlighting noteworthy findings. The method and material in section 3 delineate the proposed approach, including dataset selection, pre-processing techniques, tumor segmentation, feature extraction, and classification using both VGGNet-16 and SVM. Subsequently, section 4 experimental results and interpretation section presents detailed analyses of the performance metrics obtained from experiments conducted with the proposed methodology, offering insights into the effectiveness of VGGNet-16 and SVM models across different stages of evaluation dealing with the discussion of experimental results with the application of our study with limitation of the study and section 5 for conclusion and future prospects section summarizes our study's key findings followed with outlines potential avenues for future research to further advance early lung cancer detection methodologies.

## 2 RELATED WORKS

In the domain of medical image analysis, particularly for lung nodule detection and classification, the use of CNNs substantially advanced through various innovative methodologies. To understand the current state of lung cancer detection, it is essential to conduct a methodical review of the appropriate literature Gattasset al [39]. along with their team implemented a CNNs configuration optimized *via* Particle Swarm Optimization (PSO) algorithm. CNNs trained and validated on the LIDC-IDRI dataset, ensuring consistency across different iterations. The LIDC-IDRI dataset comprises extensive CT scans, annotated by multiple radiologists, making it a robust dataset for lung nodule research. The optimization process involved initializing a swarm of particles, each representing a potential solution for CNNs configuration. These particles explored the solution space, adjusting their positions based on individual and collective experiences, thus iteratively converging towards the optimal CNNs architecture. Their study highlighted the exceptional performance of specific test subsets, with Test-4 achieving the highest accuracy, sensitivity, and specificity, demonstrating the efficacy of the PSO algorithm in refining CNNs architectures for medical image analysis.

Sofat *et al*. [40] designed a CNNs architecture incorporating multiple ReLU and convolutional layers. ReLU activation function introduces non-linearity into the model, allowing it to learn complex patterns in the data. The convolutional layers, which apply convolution operations with learnable filters, enable the model to detect local patterns and features in the input images. Their assessment of the JSRT dataset, which contains chest radiographs, yielded noteworthy results. The multi-ReLU layers enhanced the model's capability to capture intricate features, leading to superior average metrics in terms of accuracy, overlap rate, sensitivity, and specificity. Their architecture exemplifies how deep CNNs with appropriate activation functions can effectively handle the complexity of medical image data. Incorporating ResNet principles, Wollersheim *et al*. [41] employed principles derived from ResNet for the purpose of classifying lung nodules. ResNet frameworks address the issue of vanishing gradients within deep neural networks by introducing residual blocks, enabling smoother gradient flow through shortcut connections. Transfer learning capitalizes on the feature extraction capabilities of deep networks trained on extensive datasets, resulting in notable performance enhancements despite limited medical data availability.

Additionally, they implemented curriculum learning, a strategy that entails initial training on simpler tasks before advancing to more complex ones, thereby further boosting the model's efficacy. The outcomes of their experimentation on the LIDC-IDRI dataset revealed substantial enhancements in terms of accuracy, sensitivity, and specificity. Qiang *et al*. [42]. used within the context of lung nodule detection were Deep Belief Networks (DBN). Involving various layers of Restricted Boltzmann Machines (RBMs), DBN aids in the gradual acquisition of more conceptual representations of the input data at each level. An RBM, characterized as an undirected probabilistic graphical model, is comprised of visible units and hidden units within its structure. The efficacy of their DBN-centric methodology, which was honed through training on the LIDC-IDRI dataset, is evidenced by its exceptional performance, particularly in the realm of larger nodules (>30 mm). The consistent preservation of heightened sensitivity and precision across varying nodule dimensions serves to underscore the resilience of DBNs in the domain of medical image scrutiny. Causey *et al*. [43] nodule-X unveiled as DLCNNs specifically crafted for the assessment of lung nodule malignancy through CT scans. The researchers developed intricate CNNs structures to analyze CT images from the LIDC-IDRI dataset at varying resolutions, enabling the detection of subtle features associated with malignancy. The model's efficacy was confirmed through independent validation, demonstrating notable levels of accuracy, sensitivity, and specificity, along with a remarkable AUC value, thereby emphasizing the promise of DL in the field of clinical diagnostics.

Hu *et al*. [44]. implemented a Stacked Autoencoder + Softmax strategy, integrating both 2D and 3D data for lung nodule classification. An autoencoder, is a neural network trained to reconstruct its input, consisting of an encoder that maps the input to a latent representation and a decoder that maps the latent representation back to the input space. The softmax layer, typically used for classification tasks, outputs a probability distribution over the target classes. Their approach, leveraging the LIDC-IDRI dataset, achieved outstanding results, including a minimal false positive rate and high accuracy, sensitivity, and specificity.

The integration of 2D and 3D data enabled the model to capture spatial dependencies and volumetric information, crucial for accurate lung nodule classification. In their study, Shaffie *et al*. [45] employed a Deep-Autoencoder for the non-invasive clinical diagnosis of lung nodules. Their model, trained on the LIDC-IDRI dataset, aimed to differentiate between benign and malignant nodules. A deep autoencoder, consisting of multiple hidden layers, can learn hierarchical feature representations, improving classification performance. Their results underlined promising performance metrics, with significant accuracy, specificity, and sensitivity. Wilaiprasitporn *et al*. [46]. analyzed large datasets of chest X-ray images using CNNs to detect anomalies. They assessed three retrained models across varied datasets, including ChestX-ray14 and JSRT, focusing on critical metrics such as accuracy, specificity, and sensitivity. Their study emphasized the importance of retraining models for specific tasks and datasets, with Model C, which amalgamated multiple datasets, displaying superior performance across all metrics. Bhandary *et al*. [47]. used a customized version of AlexNet for identifying lung abnormalities, including cancer and pneumonia. AlexNet, deep CNNs with multiple convolutional and fully connected layers, has demonstrated significant success in image classification tasks. Their DL methodology, applied to the Chest X-ray and LIDC-IDR datasets, achieved high accuracy, demonstrating the adaptability of AlexNet for medical image analysis.

Zhang *et al*. [48] developed a multi-view knowledge-based collaborative (MV-KBC) deep NNs model to classify lung nodules as benign or malignant on chest CT scans. This model integrates multiple views of the input data, leveraging collaborative learning to enhance classification performance. Their approach, validated on the LIDC-IDRI dataset, showed high accuracy and AUC, indicating its potential for integration into clinical workflows. Chauhan *et al*. [49] focused on enhancing lung cancer prediction using routine blood indices through various ML algorithms, including XGBoost, Logistic Regression, SVM, Decision Tree, KNN with Grid Search CV, and Gaussian Naive Bayes. Feature selection using scikit-learn algorithms retained only the most relevant features, improving model performance. Their XGBoost-based model outperformed others, demonstrating the efficacy of feature selection and ensemble learning techniques in medical prediction tasks. Ayesha *et al*. [50] made significant contributions to lung cancer type classification using feature extraction and fusion techniques, such as Discrete Cosine Transform (DCT) and patch-based Local Binary Pattern (LBP). These techniques capture texture and spatial features in chest CT scans, improving classification accuracy.

ML classifiers, including KNN and SVM, are employed to evaluate the extracted features, yielding high diagnostic precision. Li *et al*. [51]. demonstrated outstanding diagnostic precision through ML-assisted analysis of serum small extracellular vesicles (sEVs). Their approach, involving both pre-and post-surgery serum sEVs, aimed to predict tumor relapse in NSCLC patients. Ezugwu *et al*. [52] highlighted the importance of early lung cancer detection with a hybrid CNN architecture combined with the Ebola Optimization Search Algorithm (EOSA). EOSA, inspired by the Ebola virus propagation mechanism, optimizes CNN's hyperparameter, enhancing its performance. Their model, applied to the IQ-OTH/NCCD dataset, achieved high accuracy in classifying CT images, demonstrating the potential of hybrid optimization techniques in medical image analysis.

The results of various studies for cancer detection using different ML and DL models displayed offer a thorough evaluation of various parameters metrics such as accuracy, specificity, sensitivity, and F1 score/AUC detailing various datasets and sample collections consumed in each model with their outcomes from 2017 to 2023. By leveraging optimization algorithms like PSO, transfer learning, curriculum learning, and hybrid models, researchers have achieved remarkable performance metrics, paving the way for improved clinical diagnostics and patient outcomes. The integration of ML, DL, and advanced optimization techniques continues to enhance the accuracy and reliability of medical image analysis, demonstrating the transformative potential of these technologies in healthcare [53].

## 3 MATERIALS AND METHODS

This section delineates the proposed methodology integrating both DL and ML techniques. Our study utilized two distinct approaches: VGGNet-16 and SVM, conducting a comparative assessment. Fig. (**2**) illustrates our proposed method for lung cancer detection. The proposed method includes the following steps: dataset selection, pre-processing, tumor segmentation, geometric feature extraction, and classification using VGGNet-16 and SVM. In this study, the LIDC-IDRI dataset was chosen for its diversity, comprehensive annotation, and substantial sample size which makes it as a tool for advancing lung cancer detection [54]. In our study, we implemented the VGGNet-16 approach and used the libraries of Keras libraries and the Tensor Flow platform. This setup prepared the environment for developing and evaluating the VGGNet-16 model, including importing essential libraries and defining key components for data processing and model training.

### 3.1 Dataset

In the assessment of lung tissue test images, a pertinent dataset is required. LIDC-IDRI dataset identified as an ideal option meticulously curated for a thorough investigation of lung nodules on CT scans. This dataset stands out as a valuable reference, purposefully crafted to augment the capabilities of lung cancer detection and facilitate associated research endeavours which is publically available on kaggle (https://www.kaggle.com/datasets/mohamedhanyyy/chest-ctscan-image accessed on 04 March 2024). The dataset comprises three types of chest cancer: ADC, LCC, and SCC, along with a folder for normal cells. This data is organized within the main folder named Data, which contains subfolders for distinct stages of processing. The dataset's comprises training, testing, and validation, with 70%, 20%, and 10% data allocation, respectively.

### 3.2 VGGNet-16 Architecture

CNNs are specialized DL models primarily designed for processing visual data like images [5]. They excel the tasks such as image recognition, object detection, and classification [55]. In our work, the VGGNet-16 architecture begins with an input layer designed to accommodate batched RGB images of size 224x224 pixels [56]. This input processed through the pre-trained VGGNet-16 and the result is the feature maps of size 7x7x512. Following the pre-trained VGGNet-16, batch normalization applied to standardize the activation functions to maintain the stability of the model during training. Subsequently, max pooling reduces the spatial dimensions to 3x3x512, emphasizing crucial features [57]. The feature maps flattened into a 1D-vector of size 4608 and facilitate the input to a series of dense layers. These dense layers combined with dropout regularization which progressively reduce the dimensions from 1024 to 128 neurons and serve as a classifier. The final layer comprises a dense layer activated by softmax and serves as an output layer for predictions reflecting the model's assessment of lung cancer presence or absence [58]. Table **1** represents VGGNet-16 model used in our study providing a concise summary of each layer, including its type, output shape, and a number of parameters.

### 3.3 Categorization of Lung Cancer into 4 Classes Classifications

In our study, we focus on accurately classifying lung tumors into four key classes: ADC, LCC, Normal, and SCC. Each class represents a distinct type of cellular morphology in the lungs, making precise classification essential for targeted treatment. This process utilizes a two-tiered approach combining VGGNet-16 for deep feature extraction and SVM for refined classification, resulting in highly reliable diagnostic outcomes. ADC represents the most common type of non-small cell lung cancer, ADC originates in the outer regions of the lung and is commonly associated with non-smokers. Our model identifies ADC by detecting lung cancer characteristics of glandular patterns. LCC is known for its rapid growth and poor differentiation, LCC often appears in any part of the lung. The model differentiates LCC by its unique cellular appearance, devoid of specific glandular or squamous features. This class represents non-cancerous lung tissue, serving as a baseline for comparison. Our model’s ability to recognize normal lung structures ensures that benign regions are accurately classified, reducing false positives and enhancing overall reliability. Typically related to smoking, SCC originates in the bronchial tubes and features distinctive squamous cells. The model identifies SCC based on these structural patterns, enhancing specificity in diagnosing smoking-related lung cancer.

By combining the extensive feature extraction of VGGNet-16 with SVM’s discriminative capabilities, the model achieves robust classification across these four tumor types. This approach not only enhances the accuracy of each diagnosis but also contributes to targeted treatment planning by precisely distinguishing between varied cellular compositions within lung tissue.

This conclusive phase of our methodology revolves around the critical task of categorizing lung tumors into 4 classes. This pivotal step is approached through a dual-pronged strategy, integrating the strengths of both VGGNet-16 and SVM [59]. The trained VGGNet-16 model, having undergone meticulous training on an extensive dataset, is deployed to classify new and previously unseen lung tumor images [60]. Leveraging the discriminative capabilities of VGGNet-16, the model categorizes tumors based on features extracted during the training phase. This ensures robust and precise predictions, contributing to the accurate identification of all four classes [61].

### 3.4 Performance Methods

The purpose of our study is to evaluate the effectiveness of a suggested methodology using the LIDC-IDRI dataset to address the pressing need for early lung cancer identification [62]. The main objective is to create a reliable automated system that accurately classifies lung tumors in order to identify cancers in a timely manner [63]. The training of the models took place on the Kaggle platform, utilizing both its CPU and GPU resources. The storage demands for the models were around 73.1 GB, with RAM usage totaling 29 GB. Computational tasks were handled by two GPU T4 units, each providing 15 GB of memory. Our study employs the VGGNet-16 for feature extraction due to their high accuracy, sensitivity, and specificity in image-based classification. SVM operated for classification based on geometric characteristics, aiming to create an automated system for accurate lung tumor classification [64]. The mathematical expression of the performance methods are mentioned in Eqs. (**1** to **4**) [65, 66].


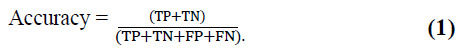



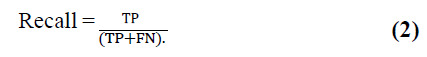



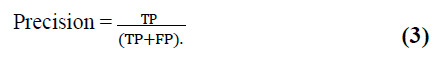



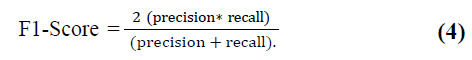


where, TP for True Positive, TN for True Negative, FP for False Positive and FN for False Negative.

### 3.5 VGGNet-16 based Analysis

VGGNet-16 was chosen over VGG-19, AlexNet, and GoogleNet for lung cancer detection due to its balanced depth, efficiency in feature extraction, robust transfer learning capability from ImageNet, manageable computational demands, and extensive community support, making it ideal for medical image analysis tasks for lung cancer detection [67, 68]. The trained VGGNet-16 model deployed for lung tumor classification, autonomously extracting intricate features from lung tumor images without explicit feature engineering. VGGNet-16 deeply handles the complexity and nuanced characteristics within lung tumor images, enabling accurate categorization [69]. This integration serves as a cornerstone in automating the categorization process, providing invaluable insights for medical diagnosis and treatment planning [70].

In our proposed methodology for detecting lung cancer, our innovative SVMVGGNet-16 method synergizes the inherent strengths of VGGNet-16 and SVM. For our study, we selected SVM for lung cancer detection because of its strong performance in high-dimensional spaces, robust resistance to overfitting, ability to handle non-linear classification effectively, and its proven reliability in medical image analysis [71]. These attributes make SVM, a superior choice compared to other ML models like Random Forest [72] and AdaBoost [73]. By capitalizing on SVM's discriminative prowess and VGGNet-16’s ability to autonomously extract complex features, our proposed model architecture and methodological stages collectively constitute a robust framework. This framework not only advances the field of lung cancer detection but also fosters in-depth research endeavors, positioning our study at the forefront of cutting-edge methodologies in medical image analysis [74]. The seamless integration of our two models augments the reliability, accuracy, and efficiency of lung cancer identification, underscoring the significance of a multifaceted approach in addressing the intricacies of medical image classification.

## 4 RESULTS AND DISCUSSION

In our experimental analysis, we evaluated the performance of VGGNet-16 and SVM models across three phases: training, validation, and testing. Then we evaluated the SVMVGGNet-16 model performance for classification of lung tumors across various 4 classes with their performance metrics.

### 4.1 Pre-processing of Dataset

A median filter is employed to reduce noise in the images, thereby enhancing image quality [75] and facilitating the straightforward identification of lung tumors. The median filter replaced each pixel's value with the median value of the intensities in its neighborhood, effectively reducing salt-and-pepper noise while preserving edges. Image contrast and sharpness improved using histogram equalization, which enhances the visibility of lung tumors. Histogram equalization redistributes the intensity values of the image, enhancing contrast by spreading out the most frequent intensity values [76].

### 4.2 Segmentation of Lung Tumors

The application of image thresholding is employed to separate lung tumors from the adjacent tissue, facilitating the identification of tumor regions. This methodology entails establishing a specific threshold value, where pixel values above it are categorized as tumor and those below it are categorized as background. The implementation of edge detection algorithms, such as the renowned Canny edge detector, is employed to identify the precise boundaries of the segmented tumors, resulting in a more precise delineation. The Canny edge detector undertakes a multi-step process encompassing noise reduction, gradient computation, non-maximum suppression, and edge tracking through hysteresis.

### 4.3 Extraction of Geometric Parameters

The area of segmented tumor regions is determined following the same procedure [77], which provides insights into tumor size. The perimeter of tumor regions is quantified to characterize their shape [77]. The eccentricity is computed to ascertain the elongation of tumors [77]. The compactness of the gauge tumor is also calculated following the same procedure [77], indicating proximity to a circular form. The circularity of the tumor was evaluated for geometric classification [77].

### 4.4 Feature Extraction from Trained Lung Cancer Images

A diverse dataset of lung cancer images, including ADC, LCC, SCC, and normal cells, was compiled. Images are standardized through pre-processing and meticulously labeled. Random sampling ensures a varied subset for robust feature extraction and facilitates comprehensive model training [78]. Employing a pre-trained VGGNet-16 model, the hierarchical features are extracted from chest cancer images and normal cells. VGGNet-16 processes images and generates the feature maps capturing specific textures, shapes, and patterns. Max-pooling layers reduce the dimensionality focusing on discriminative features. Extracted features serve as the inputs of the pre-trained VGGNet-16 model for classification [79].

### 4.5 Training Phase

In our experimental analysis of training phase, Table **2** and Fig. (**3**) illustrate the performance of VGGNet-16, SVM, and SVMVGGNet-16 models in classifying instances, particularly focusing on positive cases. VGGNet-16 exhibit remarkable accuracy and high recall, indicating their adeptness in correctly classifying positive instances. However, the relatively lower AUC value raises concerns about their ability to effectively differentiate between positive and negative cases. Conversely, SVMVGGNet-16 demonstrated a training accuracy (97.22%) and a higher AUC (0.9922), suggesting robust classification based on geometric features. Despite slightly lower recall, SVM achieves a higher F1-score (94.48%), underscoring its effectiveness in capturing positive instances accurately.

### 4.6 Validation Phase

In the validation stage of our work, Table **3** and Fig. (**4**) present the performance metrics of VGGNet-16, SVM, and SVMVGGNet-16 models. VGGNet-16 demonstrates an accuracy (83.33%) and an AUC (0.9285), indicating proficient classification. The recall, precision, and F1-Score (80.00%), (82.32%), and (81.08%), respectively, highlighting the model's ability to accurately identify positive instances. Conversely, SVM achieves accuracy (81.94%) and a higher AUC (0.9405), showcasing its robust classification based on geometric features whereas SVMVGGNet-16 had accuracy (94.72%), an AUC (97.87%), recall (86.67%), precision (91.49%) and F1-score (87.73%). Despite similar recall to VGGNet-16 Net, SVM yields slightly higher precision and F1-Score (85.17%) and (82.23%), respectively. These results underscore the trade-offs between different evaluation metrics and offer insights into the comparative performance of the two models. These results provide insights into the performance of each model during the validation phase, aiding in informed decision-making for model selection and refinement [80].

### 4.7 Testing Phase

In the testing stage of our work, Table **4** and Fig. (**5**) outline the performance metrics of VGGNet-16, SVM, and SVMVGGNet-16 models. VGGNet-16 exhibits an accuracy (84.13%) and an AUC (0.9537), demonstrating proficient classification. The recall, precision, and F1-Score (81.13%), (86.31%), and (83.58%), respectively, indicate the model's ability to effectively identify positive instances. Conversely, SVM achieves an accuracy (84.44%) and a higher AUC (0.9684), showcasing robust classification based on geometric features. Despite similar recall to VGGNet-16, SVM yields slightly higher precision and F1-Score (85.30%) and (84.56%), respectively whereas SVMVGGNet-16 achieved accuracy (96.72%), AUC (96.87%), recall (84.67%), precision (87.40%) and F1-score (85.73%) respectively. These results offer insights into the comparative performance of the two models during the testing phase, aiding in informed decision-making for model selection and deployment.

Figs. (**3**, **4**, and **5**) provide an extensive assessment of DL model used in the detection of lung cancer throughout the training, validation, and testing stages. Throughout all phases, VGGNet-16 consistently outperforms SVM in AUC, highlighting their efficacy in distinguishing between positive and negative instances in detecting lung cancer. The improvement in AUC from training to testing phases for the VGGNet-16 underscores their generalization ability and potential for real-world applications. SVM, while competent, reveals limitations in AUC across validation and testing phases, suggesting areas for refinement to enhance practical performance [81].

On the whole, SVM exhibits an outstanding efficacy during the training phase and delivers competitive outcomes in validation and testing scenarios. SVM particularly shines in managing high-dimensional datasets and performing non-linear classification tasks. Conversely, the VGGNet-16 showcases robust generalization capabilities, rendering both models’ feasible options. Nevertheless, SVM hold slight edge in terms of precision and recall, which are crucial for minimizing false positives and negatives, especially in the realm of medical applications. Now, we go with the VGGNet-16 and the SVM proposed SVMVGGNet-16 model for the classification of lung cancer with their geometric extraction in our study.

### 4.8 SVMVGGNet-16

Fig. (**6**) illustrates the correlation between training accuracy and validation accuracy, alongside training loss and validation loss. The graph designates that training accuracy improves as epochs improvement, implying enhanced classification proficiency of the VGGNet-16 and SVM algorithm with increasing epochs. Fig. (**7**) displays the AUC and Recall of our experiment, providing further perceptions into the model's performance. Finally, the classification is done by confusion matrix and ROC curve respectively, illustrated in Fig. (**8**).

### 4.9 Classification during the Training and Testing Phase with their Performance Metrics

Tables **5** and **6** depict the assessment of performance in our study on the identification of lung cancer using VGGNet-16 and SVM classifiers, involving four classes ADC, LCC, Normal, and SCC present in the image dataset operated for both testing and training phases.

### 4.10 Comparison of Our Approach

In our study, analysis conducted in Table **7** and Fig. (**9**) delineates the performance measures of VGGNet-16, SVM, and the SVMVGGNet-16 model in the context of lung cancer detection throughout both the training and testing phases. Meanwhile, Fig. (**9**) visually depicts the comparative performance of each model used in our study, VGGNet-16, SVM, and SVMVGGNet-16 models. Clearly, our SVMVGGNet-16 model achieves the highest precision rates of 97.22% during training and 96.72% during testing, emphasizing its superior effectiveness. VGGNet-16 showcases notable generalization capabilities, whereas SVM excels in managing high-dimensional data and executing non-linear classification tasks proficiently. By synergistically integrating these models within a hybrid framework, the reliability and accuracy are heightened, leading to a robust approach for diagnosing lung cancer. The comprehensive performance metrics pertaining to various categories ADC, LCC, Normal, and SCC accentuate the efficacy of each individual model.

### 4.11 Comparison with Previous Work

The progression of lung cancer classification models applied to the LIDC-IDRI dataset throughout various years (Table **8**). Commencing in 2017 with the implementation of ResNet Principles resulting in accuracy (89.9%), there had been a series of advancements leading to increasingly precise models. The year 2019 saw the introduction of Deep Autoencoder model achieving accuracy (91.20%), succeeded by MV-KBC Deep Neural Network Model in 2022 with accuracy (91.60%). In our present study, we introduced the SVM and VGG-16 Net model, showcasing the highest accuracy (96.72%) among all models listed, and signifying notable progress in lung cancer classification. This progression highlights the ongoing refinement and efficacy of DL methodologies in tasks related to the analysis of medical images.

The findings of our study regarding the detection of lung cancer through the utilization of VGGNet-16 and SVM model notable implications for clinical practice, research, and have public health endeavors. By incorporating these models into clinical processes, healthcare professionals can avail themselves of automated assistance in precisely detecting lung tumors from CT scan images, which may result in earlier diagnoses and enhanced patient results. Besides, the inception of computer-guided diagnostic systems that harness these models can enhance diagnostic accuracy and efficiency, ultimately benefiting patients by decreasing diagnostic errors. Additionally, our research contributes to growth in the context of medical image analysis and DL, establishing a basis for further exploration into the detection of lung cancer and customized cure approaches. The implementation of automated detection systems in public health for screening initiatives has the capacity to streamline early detection efforts on a population level, facilitating prompt interventions for individuals at high risk and ultimately enhancing outcomes related to lung cancer on a wider scale [82].

### 4.12 Limitation of the Study

While the study offers valuable insights into lung cancer detection using VGGNet-16 and SVM models, several limitations should be acknowledged. Firstly, the study's reliance on a single dataset, such as the LIDC-IDRI dataset, may boundary the generalized ability of the outcomes to diverse patient inhabitants or imaging protocols. Also, our study's concentration on CT scan images may overlook the potential benefits of incorporating other imaging modalities, such as MRI or PET scans, which could provide complementary information for a more comprehensive diagnosis. Furthermore, the performance of VGGNet-16 and SVM models may be influenced by factors such as the worth of image pre-processing techniques, the selection of model hyper parameters, and the availability of labeled data for training [83]. Moreover, the study primarily evaluates model performance in terms of accuracy, AUC, recall, precision, and F1-score, neglecting other important aspects such as computational efficiency or interpretability, which are crucial for real-world implementation. Finally, our study's retrospective design may limit its ability to prospectively validate the developed models in clinical settings, warranting further validation studies to assess their real-world utility and impact on patient outcomes.

### 4.13 Model Effectiveness to Demonstrate the Advantage

The following is a systematic exposition, tailored to our research, regarding the detection of lung cancer across four distinct categories:

- The comparative evaluation of our SVMVGGNet-16 model's efficacy against established methodologies will facilitate the validation of its dependability across diverse lung cancer scenarios, thereby ensuring that metrics such as accuracy, recall, and precision align with the exigencies of real-world clinical practice.
- Through the process of our novelty model, we are able to delineate the areas in which our model demonstrates superior performance, particularly in the identification of early-stage lung cancer nodules, which may enhance the early detection of cases and subsequently improve patient prognoses.
- Our novelty conducting the relative to alternative methodologies can illuminate any deficiencies in performance, thereby enabling us to refine the model and gain insights into specific domains—such as image resolution or noise mitigation—where adjustments may requisite.
- By evaluating the model in conjunction with competing methods, we illustrate its potential applicability within clinical environments, providing a holistic perspective on its benefits in practical use, ranging from accuracy to operational feasibility [84].
- Transparent benchmarking affords healthcare practitioners and researchers a lucid understanding of the model’s strengths and weaknesses [28], thereby fostering confidence in its implementation for lung cancer detection.
- Furthermore, our method serves to establish a normative standard, providing a foundational reference for prospective improvements or modifications of our model in response to the evolving landscape of diagnostic requirements [27].

This structured analysis emphasizes the particular mechanisms through which benchmarking can fortify the impact of our model and enhance its preparedness for clinical utilization.

### 4.14 Application of This Study:

The findings of our research present numerous technical applications within clinical and healthcare settings:

- The SVMVGGNet-16 model represents a robust instrument for radiologists, facilitating the expedited classification of lung cancer variants with augmented diagnostic accuracy [85]. Through the automation of the identification and categorization of specific lung cancer variants—ADC, LCC, SCC, and normal tissues—the model offers considerable assistance for initial evaluations, reducing the likelihood of human error and accelerating diagnostic processes.
- The incorporation of our SVMVGGNet-16 model into automated diagnostic frameworks can promote the early identification of lung cancer, particularly within populations at elevated risk. By embedding this system into hospital infrastructures and specialized oncology screening centers, healthcare practitioners can substantially enhance the diagnostic timeline, enabling prompt intervention and treatment, which is essential for improving survival rates among lung cancer patients [86].
- The synthesis of the VGGNet-16 and SVM in the context of lung cancer detection underscores the tangible influence of DL and ML methodologies on medical imaging practices [87]. This hybrid model functions as an instructive exemplar for medical trainees and practitioners who are keen on sophisticated diagnostic technologies, illustrating how DL can be applied in pragmatic clinical settings while bridging the educational divide between data science and medical practice.
- Upon the completion of further validation and clinical investigations, the proposed model possesses the capacity to be seamlessly integrated into conventional clinical workflows [88]. Such incorporation could enable a high-caliber, economically viable strategy for lung cancer screening and diagnosis, thereby assisting healthcare teams in achieving timely and precise diagnoses with minimal interruptions.

By establishing a framework for accurate, automated lung cancer detection, our study significantly contributes to the technological advancement of diagnostic medicine. The SVMVGGNet-16 model illustrates a promising trajectory for the integration of AI-augmented diagnostic systems within clinical practice, ultimately fostering enhanced patient care and favorable therapeutic outcomes through timely intervention.

## CONCLUSION AND FUTURE PROSPECTS

In conclusion, our study presents a comprehensive analysis of lung cancer detection developing VGGNet-16 and SVM, leveraging the LIDC-IDRI dataset. Our experimental results underscore the effectiveness of DL techniques in early diagnosis, with VGGNet-16 outperforming SVM in accuracy and AUC. Notably, our proposed model VGGNet-16and SVM achieved an impressive accuracy (97.22%) in the training phase and (96.72%) in the testing phase, representing better performance in loss minimization. These results emphasize the essential role of advanced DL methods in addressing the critical need for timely lung cancer detection. Moving forward, optimizing the VGGNet-16 training phase and refining SVM's generalization capabilities are key research directions. Fine-tuning model parameters, exploring diverse architectures, and augmenting dataset diversity can enhance VGGNet-16 discriminative capabilities. Additionally, employing hybrid methods and advanced feature engineering techniques may improve SVM's performance. Integrating explainability techniques into models could enhance interpretability, aiding in understanding classification decisions. Continuous validation on diverse datasets and collaboration with medical experts are essential for real-world applicability and reliability. In essence, our study lays a solid foundation for advancing lung cancer identification, contributing to improved initial diagnosis and patient consequences.

As for forthcoming research directions, optimizing VGGNet-16 and SVM performance during the training phase should be a focal point to enhance discriminative capabilities.

- Further refine the hybrid model by exploring other DL architectures and advance SVM kernels to enhance the detection, accuracy, and robustness.
- Incorporate additional datasets with diverse lung cancer types and varying imaging conditions to improve models' generalizability and performance across different populations.
- Conduct extensive clinical trials and collaborate with healthcare professionals to integrate the proposed model into clinical workflows, ensuring practical usability and effectiveness in medical settings.
- Develop detection capabilities, supporting automated systems to assist radiologists and oncologists in building quick and accurate diagnostic decisions during routine screenings.
- Design and implement user-friendly interfaces for automated detection systems, making them accessible and easy to use for medical consultants without specialized technical knowledge.

## Figures and Tables

**Fig. (1):**
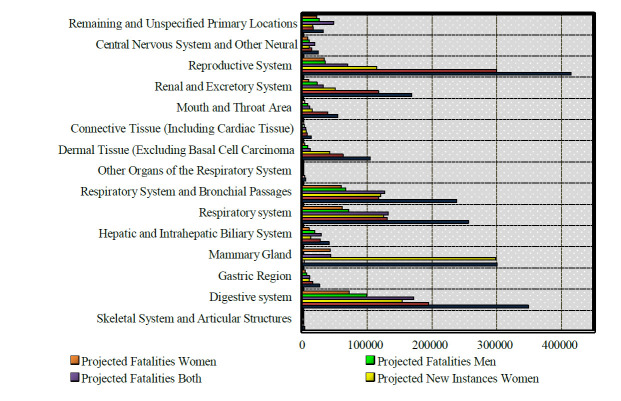
Anticipated incidence and mortality of novel cancer cases in 2023, in accordance with the NAACCR.

**Fig. (2):**
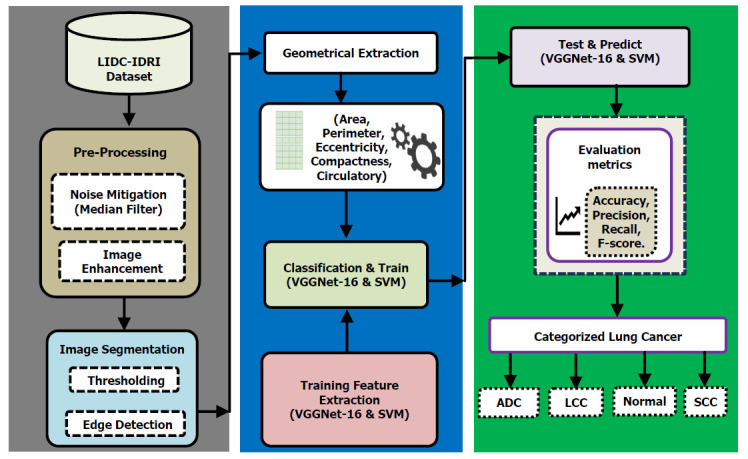
Organizational diagram of the proposed model.

**Fig. (3):**
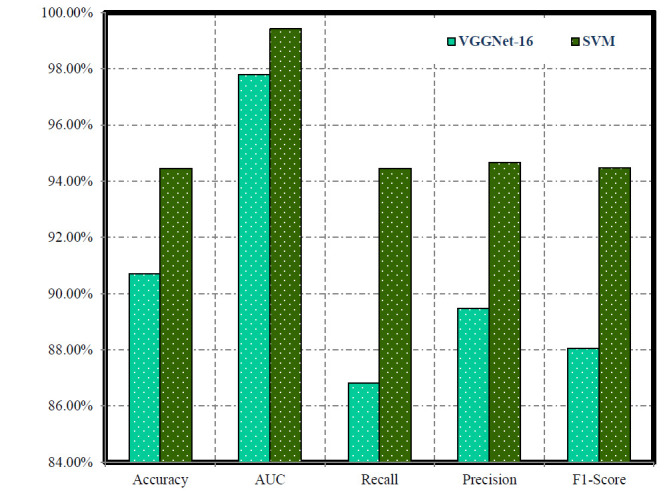
Evaluating the effectiveness of VGGNet-16 and SVM models during training phase.

**Fig. (4):**
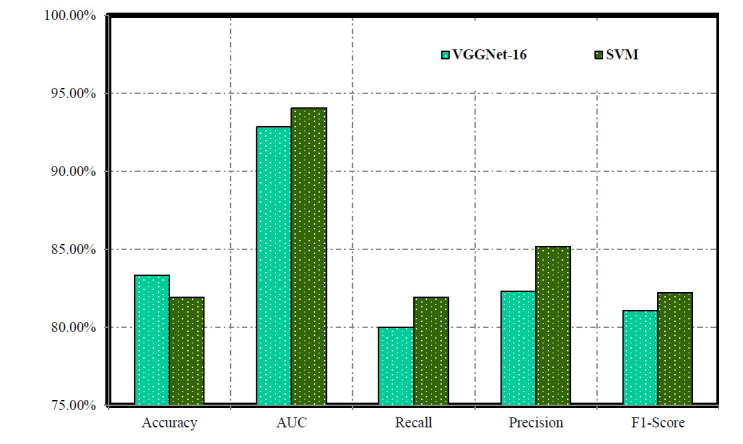
Evaluating the effectiveness of VGGNet-16 and SVM models during the validation phase.

**Fig. (5):**
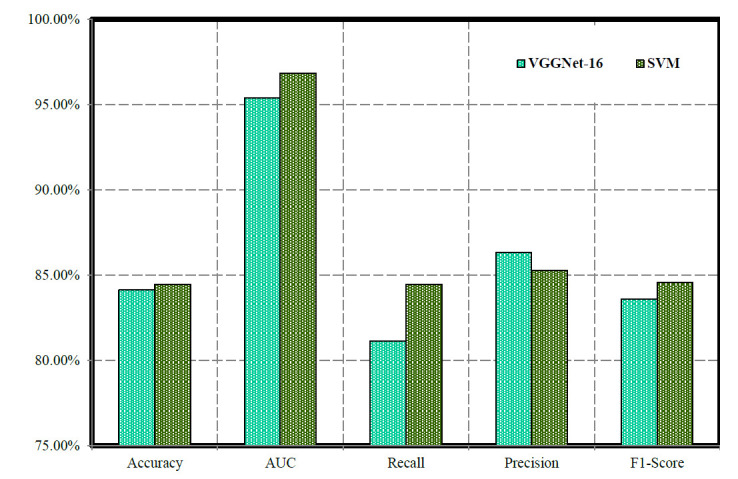
Evaluating the effectiveness of VGGNet-16 and SVM models during testing phase.

**Fig. (6a, b):**
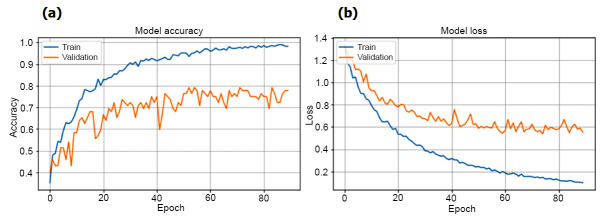
Model accuracy and model loss graph.

**Fig. (7a, b):**
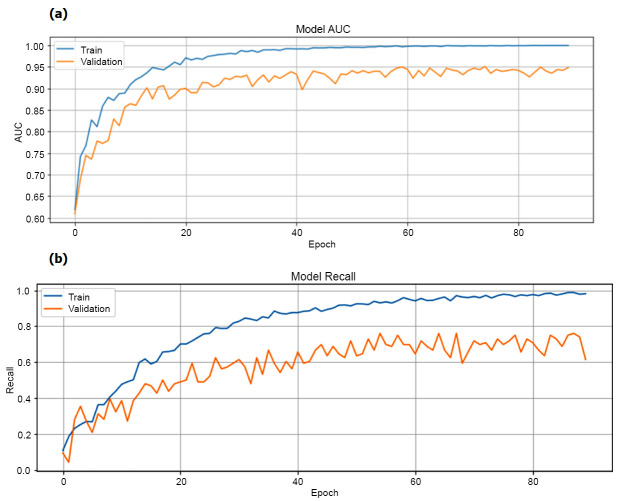
Model AUC and model recall graph.

**Fig. (8a, b):**
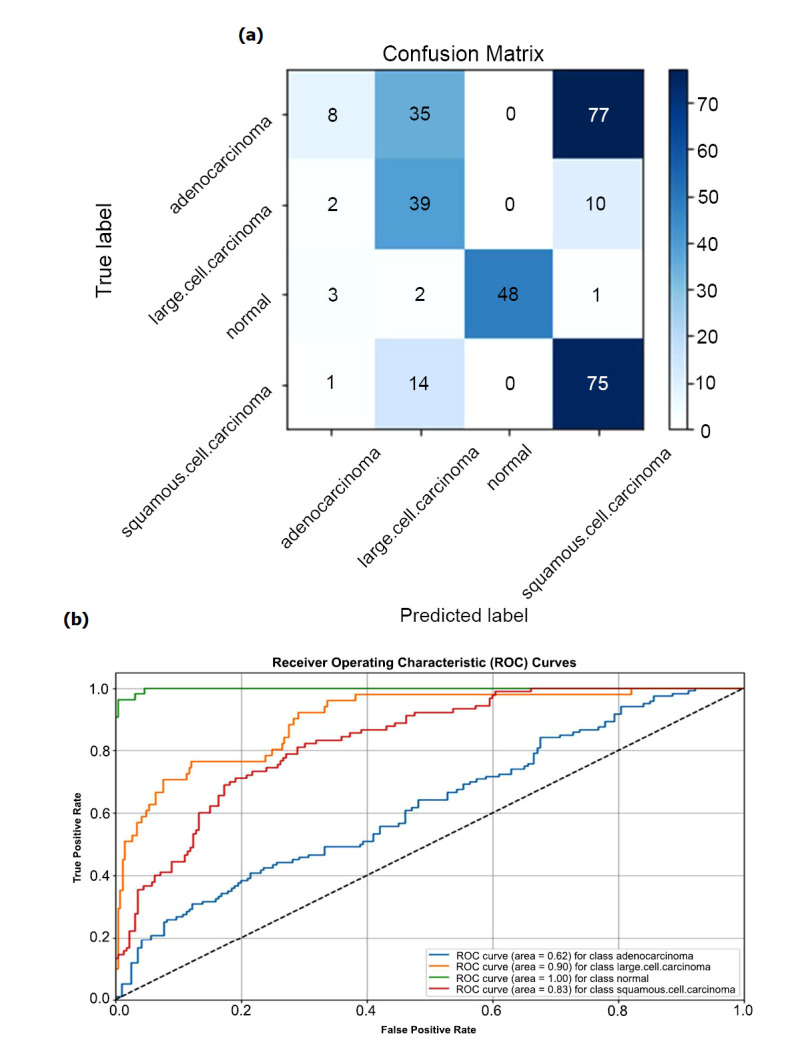
Confusion matrix and ROC graph for the classification of Lung cancer in 4 classes.

**Fig. (9):**
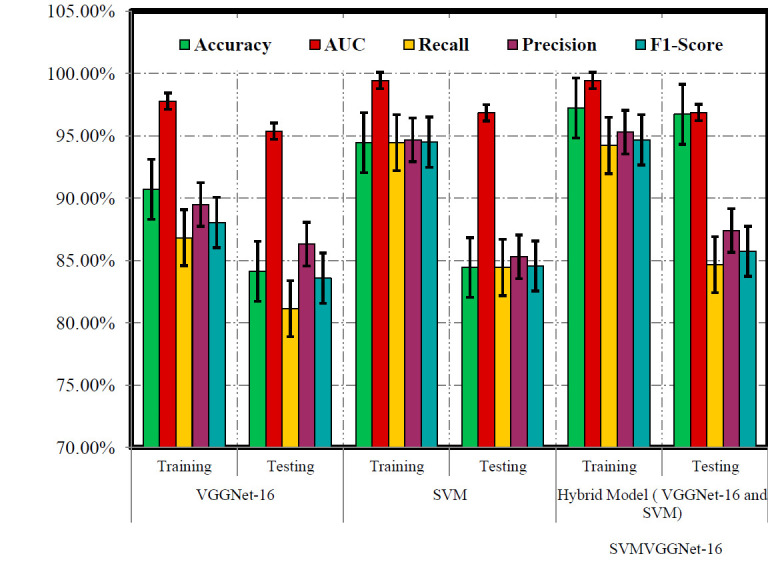
Performance of various models used in the proposed work.

**Table 1:** Representation of our VGGNet-16 model used in our study.

**Layer (Type)**	**Output Shape**	**Parameter**	**Activation**
VGGNet-16(Functional)	(None, 7, 7, 512)	14,714,688	-
Batch Normalization	(None, 7, 7, 512)	2,048	-
Max Pooling2d	(None, 3, 3, 512)	0	-
Flatten	(None, 4608)	0	-
Dense	(None, 1024)	4,719,616	ReLU
Dropout	(None, 1024)	0	-
Dense 1	(None, 512)	524,800	ReLU
Dropout 1	(None, 512)	0	-
Dense 2	(None, 256)	131,328	ReLU
Dense 3	(None, 128)	32,896	ReLU
Dense 4	(None, 4)	516	Softmax

**Table 2:** Results acquired in the training stage.

**Models**	**Accuracy**	**AUC**	**Recall**	**Precision**	**F1-Score**
**VGGNet-16**	90.70%	97.78%	86.82%	89.48%	88.04%
**SVM**	94.45%	99.42%	94.45%	94.67%	94.48%
**SVMVGGNet-16**	97.22%	99.42%	94.22%	95.28%	92.68%

**Table 3:** Results acquired in the validation stage.

**Models**	**Accuracy**	**AUC**	**Recall**	**Precision**	**F1-Score**
**VGGNet-16**	83.33%	92.85%	80.00%	82.32%	81.08%
**SVM**	81.94%	94.05%	81.94%	85.17%	82.23%
**SVMVGGNet-16**	94.72%	97.87%	86.67%	91.40%	87.73%

**Table 4:** Results acquired in the testing stage.

**Models**	**Accuracy**	**AUC**	**Recall**	**Precision**	**F1-Score**
**VGGNet-16**	84.13%	95.37%	81.13%	86.31%	83.58%
**SVM**	84.44%	96.84%	84.44%	85.30%	84.56%
**SVMVGGNet-16**	96.72%	96.87%	84.67%	87.40%	85.73%

**Table 5:** Performance measurements for our experiment during the training phase.

Class	**Type of Lung Cancer**	**Recall**	**Precision**	**Accuracy**	**AUC**	**F1 Score**
**0**	ADC	0.953846	0.898551	0.95106	0.994205	0.925373
**1**	LCC	0.913043	0.990566	0.982055	0.994205	0.950226
**2**	Normal	0.972973	0.993103	0.991843	0.994205	0.982935
**3**	SCC	0.929032	0.929032	0.964111	0.994205	0.929032
Average		0.942223	0.952813	**0.972267**	0.994205	0.946892

**Table 6:** Performance measurements for our experiment during testing phase.

Class	**Type of Lung Cancer**	**Recall**	**Precision**	**Accuracy**	**AUC**	**F1 Score**
**0**	ADC	0.875000	0.772059	0.963968	0.968799	0.820312
**1**	LCC	0.882353	0.849057	0.975556	0.968799	0.865385
**2**	Normal	0.851852	1.000000	0.984603	0.968799	0.920000
**3**	SCC	0.777778	0.875000	0.944762	0.968799	0.823529
Average		0.846740	0.874020	**0.967220**	0.968790	0.857300

**Table 7:** Study performance metrics for VGGNet-16, SVM, and SVMVGGNet-16 model.

**Model**	**Phase**	**Accuracy**	**AUC**	**Recall**	**Precision**	**F1-Score**
**VGGNet-16**	Training	90.70%	97.78%	86.82%	89.48%	88.04%
	Testing	84.13%	95.37%	81.13%	86.31%	83.58%
**SVM**	Training	94.45%	99.42%	94.45%	94.67%	94.48%
	Testing	84.44%	96.84%	84.44%	85.30%	84.56%
**SVMVGGNet-16**	Training	97.22%	99.42%	94.22%	95.28%	94.68%
	Testing	96.72%	96.87%	84.67%	87.40%	85.73%

**Table 8:** Comparison of our works with previous work.

**Year**	**Dataset**	**Model**	**Accuracy (%)**
2017 [13]	LIDC-IDRI dataset	ResNet Principles	89.90
2019 [18]	LIDC-IDRI dataset	Deep Autoencoder	91.20
2022 [21]	LIDC-IDRI dataset	MV-KBC Deep Neural Network Model	91.60
Current Work	LIDC-IDRI dataset	SVMVGGNet-16	**96.72**

## Data Availability

The data used in this study are openly available on Kaggle (https://www.kaggle.com/datasets/mohamedhanyyy/chest-ctscan-image).

## LIST OF ABBREVIATIONS

CNNs: Convolutional Neural Networks
SVM: Support Vector Machines
ADC: Adenocarcinoma
LCC: Large Cell Carcinoma
SCC: Squamous Cell Carcinoma
NAACCR: North American Association of Central Cancer Registries
ACS: American Cancer Society
DBN: Deep Belief Networks
MV-KBC: Multi-view Knowledge-based Collaborative
DCT: Discrete Cosine Transform
LBP: Local Binary Pattern
EOSA: Ebola Optimization Search Algorithm
